# When Assessment Theory Meets Generative AI: Reimagining SBA Design in Medical Education

**DOI:** 10.5334/pme.2033

**Published:** 2026-03-12

**Authors:** Nora Al-Shawee, Gerry McElvaney, Judith Strawbridge, Muirne Spooner

**Affiliations:** 1 Department of medicine, School of Medicine, Royal College of Surgeons in Ireland, IE; 2 Head of school of medicine, Royal College of Surgeons in Ireland, IE; 3 School of Pharmacy and Biomolecular Sciences, Royal College of Surgeons in Ireland, IE; 4 Royal College of Surgeons in Ireland, IE

## Abstract

Current evaluations of generative artificial intelligence (GenAI) in item-writing within medical education often concentrate on isolated dimensions such as validity or reliability, overlooking the broader theoretical foundations that underpin a trustworthy assessment design. This narrow emphasis risks oversimplifying GenAI’s role and obscuring how its adoption reshapes the relationship between quality, efficiency, and educational value. To address this complexity, this paper presents the Co-Created SBA Design (CCSD) framework, which reconceptualises assessment theory for the GenAI era through the lens of Van der Vleuten’s Utility Index. The framework offers a coherent structure for integrating GenAI into Single Best Answer development, maintaining equilibrium across the Utility Index dimensions while redefining collaboration among educators, higher education institutions, and GenAI, a technological partner that enriches the item-writing process. Within this triadic model, each contributor plays a distinct yet complementary role in sustaining assessment quality. Collectively, their interaction ensures that validity, reliability, educational impact, acceptability, and cost-efficiency remain balanced, supporting both educational integrity and sustainable innovation in medical education.

## Introduction

Single Best Answer (SBA) or multiple choice questions (MCQ) testing remains one of the most efficient forms of written assessment, offering both reliability and validity through broad content coverage and standardised administration [1,2]. The craft of writing high-quality items has traditionally relied on the educator’s ability to balance clinical authenticity, cognitive demand, and linguistic precision in a process that is as intellectually demanding as it is time consuming, a recognised barrier to producing high-quality item [3].

Amid these challenges, generative artificial intelligence (GenAI) has rapidly entered assessment workflows and the item-writing process [4]. The appeal of this new technology is clear: it offers speed and scalability. To evaluate the effectiveness of generative artificial intelligence (GenAI) in item-writing, much of the existing literature has focused on improvements in one or two dimensions of assessment, such as validity, reliability, or item-writing time [5,6,7]. While these are important gains, such a limited view risks oversimplifying GenAI’s broader influence, as assessment quality is inherently complex and multidimensional.

To conceptualise this complexity, Van der Vleuten’s Utility Index provides a comprehensive theoretical framework for understanding assessment quality, defining assessment value as the result of dynamic interaction among five interdependent dimensions: validity, reliability, educational impact, acceptability, and cost-efficiency [8]. Each is essential to the overall utility of an assessment system. These dimensions do not simply add up in a linear fashion; rather, they interact multiplicatively, meaning that a weakness in any single domain can compromise the overall quality of assessment, while multiple areas of weakness incrementally impair assessment integrity. Thus, GenAI’s promise of speed and scalability could, if not managed carefully, inadvertently weaken other domains. Understanding how GenAI influences each dimension, and how they interact, is therefore critical to integrating AI into item-writing in a way that preserves educational integrity and trustworthiness.

To address this complexity, this Eye Opener introduces the Co-Created SBA Design (CCSD) framework. The CCSD model offers a structured approach that preserves the dynamic balance among Van der Vleuten’s Utility Index dimensions while conceptualising an expanded partnership among Institutions, educators, and GenAI-a third collaborator that adds new depth to the item-writing process. Within this dynamic and holistic system, each partner contributes uniquely yet interdependently to sustaining the quality of SBA assessment. The framework delineates responsibilities that define *what* must be done, while the dimensions of the Utility Index clarify *why* these responsibilities matter. Mapping these elements together illustrates how the three pillars, institutional governance, educator expertise, and GenAI capability interact, both directly and indirectly, to uphold validity, reliability, educational impact, acceptability, and cost-efficiency in AI-assisted item-writing.

## Understanding the Utility Index in the Context of GenAI-Assisted item-writing: Defining the Lens

To appreciate how GenAI shapes SBA assessment quality, it is helpful to examine it through Van der Vleuten’s Utility Index. Each dimension within the index presents distinct considerations, yet they are intrinsically interconnected, with overall quality determined by their dynamic interplay. For example, a test composed of few items may display limited reliability, i.e. results may not be reproducible [9]. However, if that same test is used as a low-stakes SBA within a programmatic assessment framework, its educational impact may give it substantial value within the Utility Index [10]. In the GenAI context, GenAI’s capacity to generate large numbers of items quickly can increase cost-efficiency and consistency (reliability). Table 1 summarises each dimension and illustrates how GenAI integration can influence assessment quality in SBA development. While these challenges are well recognised in human-authored items [11]; without expert oversight, issues such as misalignment, variability in difficulty, or superficial reasoning can persist or be amplified due to curriculum-agnostic generation, prompt sensitivity, and reliance on generalised patterns. On the other hand, within the CCSD model, educator expertise, institutional guidance and training —as with traditional item writing [12,13] — are considered essential, co-existing concepts to GenAI where its application in assessment depends on the skill with which it is used, optimising validity, cognitive depth, and overall assessment quality.

**Table 1:** Applying Van der Vleuten’s Utility Index and its application to GenAI in SBA item-writing.


UTILITY DIMENSION	DEFINITION	POTENTIAL CHALLENGES WITH GENAI CREATED ITEMS	POTENTIAL BENEFIT OF GENAI CO-CREATED MODEL

Validity	Validity refers to the extent to which an assessment accurately measures what it is intended to measure. It is not a singular property, but a collection of interrelated evidences supporting score interpretation.	GenAI-created items may lack alignment with curricular objectives or default to recall-level tasks instead of reasoning [5].	Structured prompts designed by educators ensure alignment with curricular blueprints and cognitive levels, while institutional oversight provides quality assurance.

Reliability	Reliability refers to the consistency and stability of assessment outcomes. It ensures that results are reproducible and not influenced by item flaws.	Inconsistent item difficulty, distractor quality when prompting lacks standardisation, thus GenAI co created-items can fluctuate in quality [6].	Co-created workflows standardise prompt structures and embed iterative educator feedback, producing consistent, reproducible items that strengthen internal reliability across item sets.

Educational Impact	Educational impact refers to the effect an assessment has on teaching, learning, and professional development.	GenAI-generated items may reinforce superficial learning and fail to model clinical reasoning or judgment [6]	Chain-of-thought reasoning integrated into co-created items supports learning, enabling SBAs to function as both assessment and learning tools that model clinical reasoning.

Acceptability	Acceptability reflects the willingness of stakeholders to adopt and trust the tool, i.e., refers to the trustworthiness and legitimacy of an assessment.	Trust may be undermined by concerns about bias, transparency, and academic integrity. Some faculty remain uncertain about quality and security [14,15]. Educators are faced with a learning curve with prompt engineering skill, that limits the integration.	In the CCSD model, Institutional governance and transparent policies ensure ethical compliance, while faculty training builds confidence and shared trust in the use of GenAI for assessment design.

Cost-Efficiency	Cost-efficiency is the balance between the resources required to develop the assessment content and the educational value produced. This includes time, training, oversight, and quality assurance.	High subscription fees or technical requirements for GenAI platforms may widen equity gaps making it harder for under-resourced institutions to adopt AI tools at the same pace as better-funded counterparts. Quality training in AI literacy demands time, expertise, and coordination, adding to the upfront costs [16]	Co-created systems improve efficiency over time: prompt exemplars and faculty development reduce editing workload, while institutional licensing supports more consistent access within an institution. However, equitable access, including for under-resourced institutions, depends on broader structural mechanisms such as sector-wide negotiations, national or regional consortia, or open-access educational Large Language Models [16].


## CCSD framework: the pillars

Taken together, the examples in Table 1 highlight that GenAI’s integration into item-writing is neither inherently beneficial nor detrimental; its value depends on how effectively the three pillars manage the trade-offs across the dimensions of the Utility Index. As outlined earlier, the CCSD framework translates the Utility Index into an applied model of collaboration among educators, institutions, and GenAI. The following sections examine each pillar in turn, illustrating how their complementary roles collectively uphold the Utility Index dimensions within the CCSD framework.

The CCSD framework is conceptualised through three interdependent pillars: the educator, institutions and GenAI. Each pillar contributes a distinct but complementary role in sustaining the quality of assessment, collectively maintaining balance across Van der Vleuten’s Utility Index dimensions.

**Figure 1:**
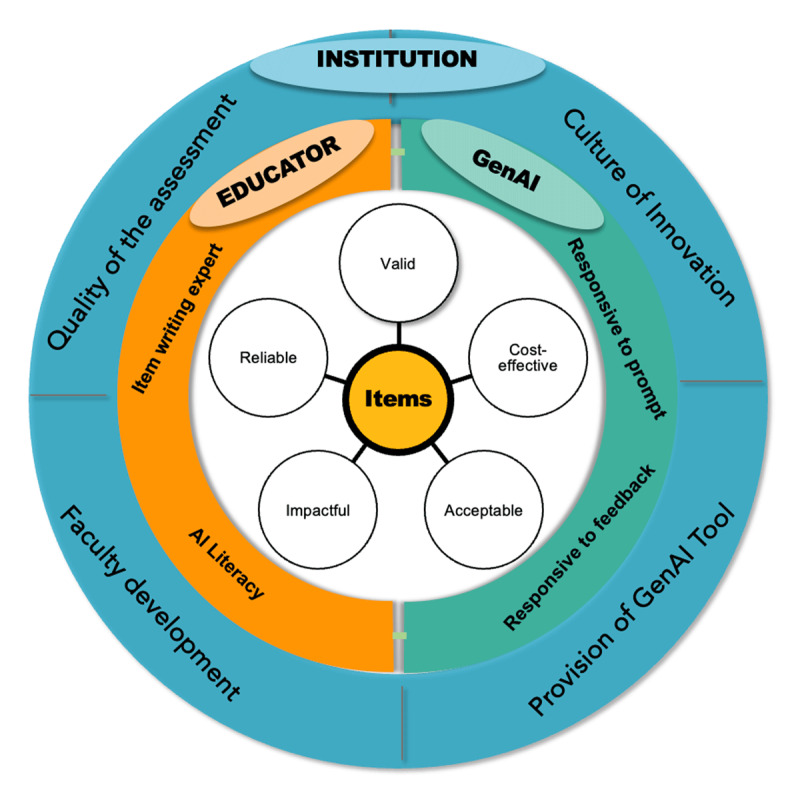
The Co-Created SBA Design (CCSD) Framework.

Figure 1 illustrates this relationship. At the centre are the assessment items, representing the product of collaboration. Surrounding them, the educator contributes academic expertise and oversight; institutions provide governance, infrastructure, and training; and GenAI adds adaptability, scalability, and efficiency. The overlapping design highlights the interdependence of all three, underscoring that no single element can uphold assessment quality in isolation.

The following sections examine each pillar in turn, explaining how their complementary roles interact to sustain balance within the Utility Index and ensure the educational integrity of GenAI-assisted SBA development.

## Pillar 1: Educators

In the CCSD, the integration of GenAI does not replace the educator’s expertise; it expands and redefines it by ensuring that GenAI-generated SBA items are designed to uphold the principles of validity, reliability, and educational value requires rethinking the educator’s role. Effective item-writing in this new landscape depends on a hybrid skill set that combines traditional item-writing expertise with the emerging AI-related competencies.

### 1. Item-writing Expertise

This traditional skill remains indispensable. GenAI can produce large numbers of items that generally align with the provided blueprint [7]. However, there is a risk that they will hallucinate (i.e., when GenAI generates content that appears plausible but is factually incorrect or entirely fabricated) and, as they are trained as large language models, can become an echo chamber of what they are “fed” [17]. These are both threats to testing authentic, real-world clinical knowledge. In order to ensure items test what they set out to test, i.e., are valid, subject matter experts must recognise and gatekeep for these limitations. Crafting high-quality SBA items that align with curriculum blueprints, engage appropriate cognitive levels, and meet psychometric standards (e.g., NBME guidelines) has always been core to assessment literacy [1]. In the GenAI context, this expertise becomes the anchor point, guiding prompt construction and detecting flawed outputs—for example, an item that tests the correct content but is not stage-appropriate, or which develops a well-constructed vignette but provides implausible distractors. Similarly, by “training” your GenAI, it is possible to develop adaptive difficulty calibration by feeding back to it, signposting stage appropriateness, providing it with vertically integrated learning outcomes of increasing complexity, and ensuring that AI-generated items are accurate, valid, and fit for purpose [18]. One of the strongest arguments for reliability in assessment is broader sampling of the construct [8]. GenAI offers significant potential here, providing a single item writer that offers theoretically consistent item-writing with scalability. However, during our extensive testing, we experienced that with the same prompt, GenAI output can vary widely in clinical accuracy, depth, and plausibility of distractors. This reaffirms the need to position the educator as an indispensable companion to GenAI, combining its ability for mass production with the educator’s expertise in curating.

### 2. AI Literacy, including prompt engineering

To use GenAI effectively in item-writing, educators must also acquire a working level of AI literacy, understanding how GenAI systems operate, their capabilities and limitations, and the ability to recognise biased or hallucinated outputs [17,19]. Without this awareness, flawed content may be accepted uncritically, compromising validity. Educators must understand how GenAI generates output, by drawing on probabilistic patterns in large datasets [20]. In contrast, human clinical reasoning is an adaptive, real-time process that integrates data, generates and tests hypotheses, and links thinking with clinical decision making [21,22]. AI literacy also underpins acceptability: educators who are unfamiliar with GenAI often express legitimate concerns about data security, intellectual property, and the legitimacy of AI-generated items. Our experience indicates that these concerns can be mitigated when institutions scaffold the item-writing process through institutional secured GenAI platforms, targeted training and the use of structured prompts, resulting in outputs that are both secure and of high quality.

Within AI literacy sits the applied competence of prompt engineering, not merely writing inputs, but understanding how prompt wording, context, and task framing shape the quality and cognitive level of AI outputs. By specifying parameters such as learner stage, clinical context, cognitive domain, and item structure, educators can direct GenAI to generate questions aligned with curricular outcomes and assessment blueprints. Conversely, poorly specified prompts can produce misaligned items or unpredictable shifts in cognitive demand (e.g., drifting from factual recall to application-level reasoning because the desired level was not stated), undermining internal consistency and threatening reliability [23]. In the context of the Utility Index, structured prompt design represents a practical mechanism for balancing multiple dimensions simultaneously. Well-crafted prompts not only enhance validity by aligning GenAI outputs with curricular blueprints and cognitive expectations, but also strengthen reliability through standardisation and reproducibility of item format. At the same time, they improve cost-efficiency by reducing post-generation editing time.

Beyond the practical dimensions of prompt design, its influence on validity warrants explicit consideration within the Utility Index framework. In assessment theory, validity concerns the extent to which an assessment measures what it intends to measure and supports appropriate interpretation of scores. In the context of GenAI-assisted item-writing, the prompt itself becomes the primary mechanism that shapes validity, it determines the content, cognitive process, and construct that the AI models. Poorly specified prompts can therefore weaken validity by producing items that test unintended knowledge domains, omit key reasoning steps, or fail to reflect curricular objectives.

In assessment research, validity is supported by multiple forms of evidence that collectively establish the credibility of score interpretation. Messick conceptualised validity as a unified construct comprising six interrelated aspects: content, substantive, structural, generalisability, external, and consequential validity [24]. When applied to GenAI-generated SBAs, studies have demonstrated varying levels of content accuracy, while substantive validity (the extent to which items engage the intended cognitive processes) examined with psychometric analyses such as item difficulty and discrimination indices, also has mixed findings: some AI-generated items performed comparably to human-written questions, whereas others showed lower discrimination indices. In many cases, these discrepancies were attributed to poorly constructed or insufficiently specific prompts. Ultimately, the validity of GenAI-items rests on the quality of its prompts, which rely on the competency of the educators engineering the prompts.

## Pillar 2: GenAI

Within the CCSD framework, GenAI functions as the technological collaborator in the co-creation process. We define this by two key capacities: responsiveness to prompts and responsiveness to feedback. The first determines how effectively GenAI interprets structured educator input, while the second reflects its ability to refine and optimise item outputs based on iterative human review. Together, these capacities influence the reliability, validity, cost-efficiency, and educational impact of GenAI-assisted SBA development.

### 1. Responsiveness to Prompts

Alongside educator expertise and institutional oversight, GenAI acts as the technological collaborator within the CCSD framework, bringing adaptability and scalability to the co-creation process [25]. A responsive system can generate items that align with curricular intent and cognitive level, thereby preserving validity. It also ensures reliability through consistent interpretation of structured prompts, thus reducing the variability between outputs [26]. This consistency contributes to greater internal consistency, as items generated under stable prompt conditions are more likely to measure the same construct consistently. In psychometric terms, such uniformity can improve indicators such as Cronbach’s alpha, reflecting higher reliability across item sets [9].

However, the same consistency that strengthens reliability also introduces potential risks. When GenAI generates items repeatedly from similar prompt structures, outputs may begin to reflect the phrasing and conceptual patterns of a single “author,” reducing content diversity and the range of reasoning processes represented. From a Utility Index perspective, this over-homogeneity may appear to enhance reliability while inadvertently diminishing educational impact, as uniform items narrow cognitive diversity and limit opportunities for higher-order reasoning. Because GenAI models are trained on large datasets that may contain cultural, gender, and contextual biases, this uniformity can also reproduce or amplify these biases, undermining fairness and inclusivity [27]. In this sense, greater reliability achieved through repetition may paradoxically undermine the educational purpose of assessment.

Within the CCSD model, balance across the Utility Index dimensions is maintained through structured oversight at both institution and educator levels. At institution level, governance can be provided by monitoring psychometric performance: analysing item difficulty, discrimination indices, and internal consistency, to ensure that GenAI-generated items uphold validity, reliability, and educational impact. Continuous monitoring allows institutions to detect patterns of over-homogeneity and recalibrate prompt design, safeguarding construct diversity and sustaining the quality of outputs. Educators, in turn, provide granular oversight through iterative feedback, recognising that GenAI models have not yet reached the level of accuracy required to generate reliable, high-stakes assessment items without human verification and refinement [28].

A key characteristic reinforcing this responsiveness, is the chain-of-thought reasoning embedded within modern GenAI systems [29]. This feature enables the model to make its reasoning steps, articulating the rationale for the correct answer and the logic underlying distractor elimination. While chain-of-thought prompting was originally developed to enhance performance in earlier instruction-tuned models (e.g., GPT-4), newer reasoning architectures (such as o3 and Gemini 2.5 Pro) now conduct internal reasoning without requiring such scaffolding. However, within the context of educational assessment, the externalisation of reasoning remains highly valuable. In formative and adaptive learning environments, particularly those using SBA assessments, structured prompts that elicit visible reasoning promote metacognition, feedback literacy, and self-explanation. These processes directly enhance educational impact, a central dimension of assessment utility as articulated by van der Vleuten (1996), “educational impact is the heart of educational achievement testing.” By supporting deeper cognitive engagement and meaningful learning from feedback, and affirming the principle that assessment drives learning [30].

Moreover, externalised reasoning contributes to acceptability and validity by making the model’s decision-making transparent and providing an inspectable rationale that educators and learners can critically appraise. Such transparency enables users to identify conceptual faults within generated items and fosters reflective engagement. For example, reviewing the model’s rationale allows users to detect hidden fault in an item on atrial fibrillation that reveals, in its rationale, an incorrect claim that rhythm control is never indicated in management. Thus, the educational value lies in prompting critical evaluation, not in accepting the model’s reasoning as truth. Although explicit chain-of-thought prompting may be redundant for reasoning models in computational terms, its continued use for instructional transparency and learner reflection strengthens the overall utility of AI-enabled assessment [8,31].

### 2. Responsiveness to Feedback

The second defining capacity of GenAI is its responsiveness to feedback: its ability to refine and adjust item outputs following human review. In this iterative process, the educator’s prompt represents the first interaction, while subsequent feedback functions as a *calibration* mechanism, guiding the model to enhance quality, cognitive alignment, and linguistic precision. This responsiveness extends beyond correction; it reflects the system’s capacity to adapt dynamically, modifying item difficulty, improving distractor plausibility, and refining content coherence in response to human oversight.

When effectively operationalised, this feedback loop directly supports several dimensions of Van der Vleuten’s Utility Index. Responsiveness to feedback promotes validity, as AI models learn to generate items more accurately aligned with curricular blueprints and cognitive taxonomies. It enhances reliability by reducing random variation and inconsistencies across outputs, and it advances cost-efficiency by progressively decreasing the time and effort required for post-generation editing. Over time, a responsive GenAI system can develop predictive accuracy in meeting educator expectations, shortening review cycles while maintaining or even improving item quality.

Importantly, feedback responsiveness also contributes to educational impact. When the model learns from iterative human input, it begins to produce richer explanations, more contextually appropriate vignettes, and better-structured reasoning pathways, features that transform assessment items into tools that support learning as well as evaluation. This responsiveness to feedback enables GenAI to move beyond automation toward genuine collaboration, where efficiency is achieved without compromising pedagogical depth and where the process itself contributes to student learning [30].

## Pillar 3: Institutional Support in the CCSD

Within the CCSD framework, academic institutions form the outer layer that enables and sustains all other functions of GenAI-assisted SBA item development. Its influence operates through four interconnected responsibilities.

### 1. Fostering a culture of innovation

Within the CCSD framework, cultivating a culture of innovation represents the first and most critical institutional responsibility, as it sets the conditions for all other dimensions of GenAI-assisted item-writing to succeed.

Implementing an innovation in assessment begins with “belief”, i.e., to achieve sustainable adoption it requires a tactical engagement with educator beliefs and values to adapt the new technology. Historically, the integration of emerging technologies within higher education has been met with hesitation, shaped by cautious stance toward rapid change [32]. Additionally, educators are often unaware of educational research or do not consider it central to their professional practice, which limits their engagement with evidence-based innovation. This disconnect means that simply introducing a new technology does not equate to educator buy-in [8]. This distinction between diffusion and active dissemination is the fundamental reason why an institutional culture of support is essential in adapting any new approach or innovation [33] . Passive diffusion assumes that exposure to new tools will naturally lead to adoption, whereas active dissemination requires institutional, structured engagement with contextualised evidence, and deliberate efforts to align innovation with existing beliefs and professional values of the educators.

In the context of GenAI-assisted item-writing, this distinction is particularly critical. Institutions must adopt an innovative role that sheds inherited hesitancy and perceive this integration as a necessity [32]. This shift in mindset therefore extends beyond merely providing access to AI platforms; it requires institutions to generate evidence that demonstrates the feasibility and cost-efficiency, such as reductions in item development time, growth of item banks, and psychometric analyses confirming that GenAI-generated items perform comparably to traditionally authored questions in validity and reliability. This approach aligns with Van der Vleuten’s recommendation to “strategically use the information on faculty and student beliefs in order to get their commitment,” building credibility and trust around innovation.

A culture of trust can be further reinforced through clear institutional policies developed with input from key stakeholders, including educators, administrators, and students [25,34]. Such inclusive collaboration ensures that guidelines are both practical and principled, articulating shared values and ethical standards for the use of GenAI in education and assessment. In doing so, the institution’s role in the CCSD model enhances transparency, reinforces professional confidence, and strengthens acceptability across the system.

### 2. Provision of the GenAI Tool

The second institutional responsibility within the CCSD framework concerns the provision of a secure and accessible GenAI platform. A wide range of AI tools are now available, varying in functionality, subscription models, and associated costs [17]. This variability in access and cost can pose substantial barriers, particularly across diverse global higher education contexts where resource availability and institutional funding differ [28]. It also cannot be presumed that individual educators will personally finance subscriptions or navigate the complexities of data agreements. Such reliance on individual educators raises issues not only related to equity and accessibility but also exposes institutions to risks around data privacy, confidentiality, and intellectual property.

Given these considerations, the responsibility for selecting and providing the AI platform must rest with the institution ensuring alignment with organisational values, regulatory requirements, and ethical standards, and assuring that the chosen system meets local data protection laws and maintains the integrity expected within higher education. While centrally managed, institution-vetted subscriptions can provide consistent access and reduce reliance on personal accounts, and we recognise that this approach does not fully address all security or privacy concerns. In such contexts, locally hosted open-source Large Language Models offer a more robust solution.

Equally important, educators may feel apprehensive about incorporating AI into their professional practice, particularly in the context of creating assessment items. An example in this context is when inputting an assessment blueprint into an AI system to generate exam questions raises fears about potential data breaches or unintended share of intellectual property. These privacy concerns may hinder AI adoption, questioning whether AI technology can be “trusted,” a recognized barrier to AI integration [28]. Such concerns highlight the need for institutions to provide AI platforms that align with institutional privacy standards, workflow requirements, and financial capacities. Institutional governance in this area not only protects sensitive assessment materials but also enhances educator acceptability and reinforces that the use of GenAI is both legitimate and secure.

### 3. Faculty development

As introduced in Pillar 1, the CCSD framework identifies two educator competencies as central to GenAI-assisted assessment design: item-writing proficiency and AI literacy. Within this framework, institutional responsibility extends beyond simply adopting new tools—it involves strengthening and evolving these competencies to meet emerging demands. The first, item-writing proficiency, remains fundamental to assessment quality and is best developed through targeted training [13,35]. The second, AI literacy, has become equally essential, as the integration of GenAI into assessment design requires educators to collaborate effectively with AI systems and critically evaluate their outputs [34,36,37,38]. Accordingly, faculty development initiatives must move beyond traditional item-writing instruction to include structured preparation for GenAI integration. Drawing on the AMEE Guide and informed by the Technological Pedagogical Content Knowledge (TPACK) framework, such programs should equip educators with the competency to use these emerging tools confidently and responsibly [28,39]. This includes developing a working understanding of GenAI tools, how to access and apply them appropriately in assessment design, recognise their limitations, and employ prompt engineering strategies.

Consequently, institutions are increasingly expected to take a proactive role in embedding prompt-engineering within broader AI literacy initiatives [38,40]. When educators apply well-structured prompts in item-writing, the resulting AI-generated outputs tend to contain fewer flaws, reducing the need for extensive post-generation editing and review [41].

Faculty development for GenAI assisted item-writing should not be viewed as a burden, but rather represents a strategic investment in educators’ expertise, enhancing the core competencies required for high-quality assessment design in the GenAI era. By explicitly modelling these frameworks while providing faculty development around GenAI assessment, institutions can leverage educators’ excitement and reduce apprehension toward technology adoption [28].

Additionally, implementing targeted training workshops provides the foundation for informed and balanced trust among educators while strengthening their GenAI literacy. Such workshops also increase educators’ interest and confidence in using GenAI within their professional practice [38]. Once trust and engagement are established, they collectively enhance the overall acceptance of GenAI integration into the item-writing process. As educators gain proficiency with GenAI, the time required to produce high-quality items decreases, enabling educators to design, evaluate, and refine prompts effectively. In this way, faculty development serves not only as a safeguard for the validity and reliability of AI-assisted assessments but also as a strategic mechanism for enhancing cost-efficiency in the long term.

To strengthen educators’ confidence and competence in using GenAI for item-writing and to enhance acceptability, the CCSD framework advocates for the active dissemination of high-quality, institutionally supported prompts rather than relying on passive diffusion. This approach includes embedding exemplar prompts within faculty development workshops, contextualising their use to local curricula, and providing ongoing institutional support to ensure consistent adoption. The rationale for this approach is based on the fact that passive diffusion alone is often insufficient; even well-designed innovations frequently fail to spread effectively in educational contexts [33]. Likewise, the increasing availability of structured and hybrid prompts for generating SBAs, does not guarantee their use [42]. In our faculty development workshops, for example, when participants were invited to share prompts previously used for item-writing, none reported applying pre-published examples, underscoring the need for more intentional dissemination and modelling within institutional practice. The second rationale for this approach draws on Vygotsky’s concept of the zone of proximal development, which conceptualises educators’ learning trajectories in GenAI-assisted item-writing. If educators’ existing item-writing skills represent the zone of achieved development, what they can do independently, then the ability to craft effective GenAI prompts lies within their zone of proximal development, achievable through structured guidance, institutional support, and exemplar modelling [43]. Providing scaffolded prompts therefore serves as an educational bridge, facilitating progression from dependence to competence. Over time, this guided approach fosters autonomy, enabling educators to design and refine prompts independently while maintaining assessment validity, reliability, and alignment with institutional standards.

At our institution, we developed and implemented a structured prompt as part of a faculty development workshop on AI-assisted item-writing (Supplementary Box 1). This prompt was intentionally designed to incorporate three key strategies: structural formatting, few-shot prompting, and chain-of-thought reasoning [44,45]. As illustrated in supplementary figure 2, the degree of contextual information provided to the LLM, shapes the alignment of output with assessment intent and overall quality of AI-generated MCQs, i.e. the item validity, reliability and educational impact.

### 4. Quality Assurance and Feedback Mechanisms

While prompt engineering plays a critical role in guiding AI-generated content, the process does not end with initial item creation. To ensure that AI-assisted SBAs meet educational standards and are suitable for formative or summative use, a robust review and feedback mechanism is essential. Even with well-structured prompts, GenAI may produce content that is factually inaccurate, poorly calibrated in difficulty, or misaligned with the intended cognitive level [46]. This feedback stage therefore acts as a safeguard across several dimensions of Van der Vleuten’s Utility Index. It strengthens validity by ensuring that items accurately reflect intended learning outcomes, enhances reliability through the consistent application of review criteria, and supports educational impact by maintaining cognitive diversity and authenticity in assessment tasks. Engaging stakeholders, particularly students, in this process further improves acceptability, by providing insight into the perceived fairness, clarity, and relevance of GenAI-generated items [47]. Finally, as educators become more proficient reviewers, the efficiency of the feedback cycle improves, enhancing cost-efficiency by reducing the need for extensive post-generation editing.

At the individual level, educators must critically appraise AI-generated items for clinical accuracy, cognitive alignment, and conceptual soundness, while identifying and correcting common flaws that frequently occur in GenAI outputs. Table 2 outlines typical types of edits required during review, and was identified through the authors’ analysis of AI-generated items, which can function as both a quality-assurance checklist and a teaching aid within faculty development workshops.

**Table 2:** Common Types of Edits Required for Reviewing GenAI-Generated Assessment Items.


TYPES OF EDITS	DESCRIPTION

Clinical inaccuracy	Incorrect or outdated medical content. For example: Suggesting oral amoxicillin as first-line treatment for hospital-acquired pneumonia.

Lack of depth or cognitive challenge	Questions that only assess factual recall. For example: “What is the normal range for potassium?” instead of applying this in a clinical context.

Implausible distractors	Options that are obviously incorrect or unrelated to the scenario. For example a clinical vignette on acute myocardial infarction with possible options: A. Acute myocardial infarction B. Acute pancreatitis C. Costochondritis D. Tension pneumothorax. Another example for implausible distractor, providing “pregnancy test” as an answer option for a male patient

Use of abbreviations	Use of abbreviations that may be ambiguous, outdated, or unfamiliar. For example: “NAD” (which may mean “no abnormality detected” or “nicotinamide adenine dinucleotide”)

Jurisdiction specific terminology	Use of terms that vary by region or practice. “Emergency room” (US) vs. “Accident & Emergency” (UK).

Region specific medication names	Differences in drug names across regions that may confuse test-takers. For example: paracetamol vs acetaminophen

Ambiguous or vague wording	Lack of specificity or clarity in the lead in question. For example: “Select the correct treatment” without clarifying whether it refers to first line, symptomatic, or emergency treatment.


## Conclusion

The rapid emergence of GenAI in SBA assessment design represents both an opportunity and a challenge for medical education. While GenAI offers clear advantages in scalability, efficiency, and item consistency, its unbalanced adoption risks eroding the very principles that underpin trustworthy assessment: validity, reliability, and educational value. Without structured oversight, AI-generated items may drift toward superficial recall, factual inaccuracies, or ethical uncertainty, threatening both the credibility and acceptability of assessment systems.

The CCSD framework offers a mechanism to restore and sustain this balance. By positioning educators, institutions, and GenAI as interdependent collaborators, the framework operationalises Van der Vleuten’s Utility Index for the GenAI era. Within this triadic structure, each partner safeguards complementary dimensions of quality.

Looking ahead, the CCSD framework provides a roadmap for responsible integration, guiding how GenAI can be meaningfully aligned with assessment theory and educational purpose. As institutions begin to embed GenAI within assessment practice, its true value will be realised not through automation, but through the thoughtful partnership between human experts.

## Additional File

The additional file for this article can be found as follows:

10.5334/pme.2033.s1 Supplementary Material. Supplementary Box 1 and Supplementary Figure 2.
