# Supplementary material for: When Assessment Theory Meets Generative AI: Reimagining SBA Design in Medical Education
Source: Perspect Med Educ. 2026 Mar 12;15(1):239–50. doi: 10.5334/pme.2033 (PMC12985870; doi:10.5334/pme.2033)
Supplement: Supplementary Material. — Supplementary Box 1 and Supplementary Figure 2. [file pme-15-1-2033-s1.pdf]

**Supplementary Material:**

1. **Supplementary Box 1:**

**Prompt for Medical MCQ Generation**

Task: Generate NBME-style Clinical Reasoning Multiple choice question (MCQ)

Persona: You are a clinical educator in the department of Medicine.

Blueprint:

Target Audience: Final year medical students

Question Type:

- Focus on clinical reasoning, such as integrating symptoms, risk factors, and investigations to arrive at a diagnosis or management decision.
- Avoid direct fact recall.

Instructions for MCQ Creation:

1. Clinical Vignette:
   - Age: Choose a realistic patient age.
   - Gender: Choose appropriate gender.
   - Setting: e.g., Emergency Department, GP clinic.
   - Presenting symptoms: Include primary complaint(s).
   - Duration of symptoms: Specify time frame.
   - Medical history: Add relevant background.
   - Physical findings: Include key examination findings.
   - Diagnostic results: Include relevant lab/imaging findings, if any.

2. Lead-In Question:
   - The question should require clinical decision making, Based on the learning outcome.
   - Specify the focus of the question (e.g., diagnosis, initial investigation, next step in management, treatment).



3. Answer Options:

- Provide 5 plausible answers (A to E), only one of which is correct.
- Distractors should be commonly confused alternatives and clinically plausible.
- Distractors should reflect common pitfalls or misinterpretation
- Each option must consist of a single, discrete clinical action or decision.
- Avoid combining interventions in a single option (e.g. in asthma management item, do not write "Give oxygen and prepare for intubation" in one option).

4. Correct Answer:
   - Clearly identify the correct option (e.g., A, B, C, etc.).

5. Explanation
   - Explain why the correct answer is correct.
   - Briefly explain why each distractor is incorrect or less appropriate.

6. Learning Outcome:
   - State what this question aims to assess based on the blueprint added.

8. Format Requirements:
   - Use British English.
   - Limit the clinical vignette to 120-150 words.
   - Explanation should be 3-5 concise sentences.

MCQ Structure:

Clinical Vignette:
A [Insert age]-year-old [Insert gender] presents to the [Insert setting] with [Insert presenting symptoms] for [Insert duration]. The patient's medical history includes [Insert history]. On physical examination, [Insert findings]. Investigations reveal [Insert diagnostic results].
Lead-In Question:
[Insert clear, focused clinical question—diagnosis, management, next step, etc.]
Answer Options:
A. [Insert Option A]
B. [Insert Option B]
C. [Insert Option C]
D. [Insert Option D]

E. [Insert Option E]
Correct Answer:
[Insert correct letter]
Explanation (Chain-of-Thought):
[Insert reasoning behind correct answer. Include 1–2 lines on why each distractor is incorrect.]
Learning Outcome:
[Insert what this question aims to assess]

Example MCQ:
A 62-year-old woman is brought to the emergency department by ambulance following a collapse on the road. Upon regaining consciousness, she reports a history of exertional chest pain, particularly when walking uphill. Her vital signs are within normal limits. On auscultation, a systolic murmur is noted at the right second intercostal space. An ECG performed in the emergency department shows evidence of left ventricular hypertrophy. Based on this clinical presentation, what is the most likely diagnosis?

A. Aortic stenosis
B. Hypertrophic cardiomyopathy
C. Ventricular septal defect
D. Atrial septal defect
E. Tricuspid stenosis

Correct Answer: A. Aortic stenosis
Explanation:
The patient presents with syncope, exertional chest pain, and a systolic murmur at the right second intercostal space—classic features of aortic stenosis. Left ventricular hypertrophy on ECG supports this due to chronic pressure overload.
• B. Hypertrophic cardiomyopathy typically presents in younger patients and the murmur varies with posture.
• C. Ventricular septal defect is congenital and usually diagnosed in childhood.
• D. Atrial septal defect presents with a fixed split second heart sound and often goes unnoticed until adulthood.
• E. Tricuspid stenosis is rare and typically presents with signs of right heart failure.
Learning Outcome:
Develop a differential diagnosis for a patient presenting with collapse, syncope, and exertional chest pain.

**2. Supplementary Figure 2:**

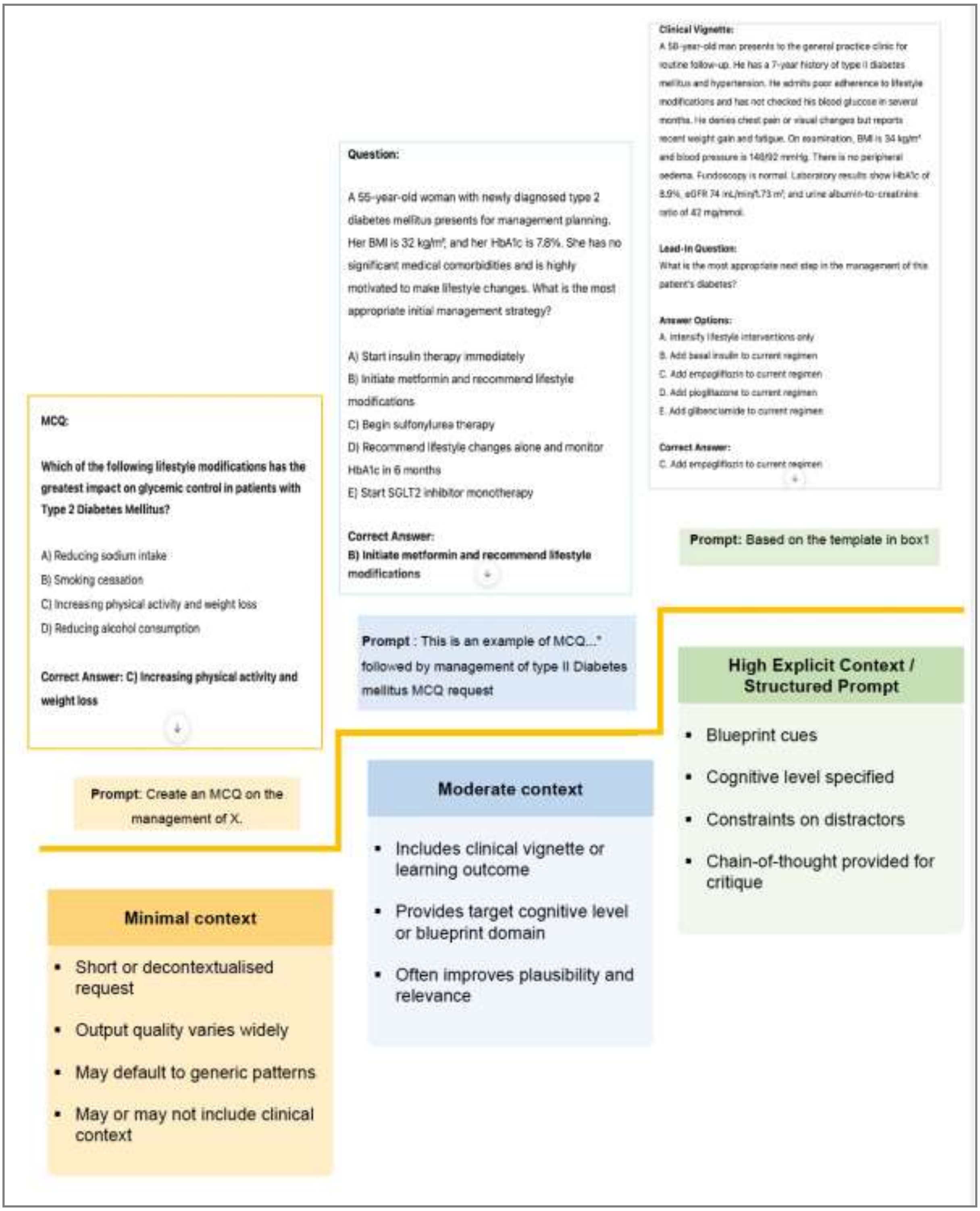


**Figure 2:** How increasing the Prompt contextual information shapes the quality and alignment of LLM-generated MCQs
